# Effects of the Loess Plateau on Habitat Quality of the West Qinling Mountains, China

**DOI:** 10.1002/ece3.71289

**Published:** 2025-05-07

**Authors:** Caihong Hui, Xuelu Liu, Miaomiao Zhang, Xiaoning Zhang, Xingyu Liu

**Affiliations:** ^1^ College of Resources and Environmental Sciences Gansu Agricultural University Lanzhou China; ^2^ College of Management Gansu Agricultural University Lanzhou China; ^3^ College of Forestry Gansu Agricultural University Lanzhou China

**Keywords:** habitat quality, influencing mechanism, land use, spatial and temporal difference, the west Qinling Mountains

## Abstract

The West Qinling Mountains is the western extension of the Qinling Mountains, the geographic demarcation line between north and south China. Under the control of the Loess Plateau, the northern part of the West Qinling Mountains has obvious transitional features in terms of topography, climate, soil, and vegetation. In order to explore the effects of the Loess Plateau on habitat quality (HQ) of the West Qinling Mountains, we selected five typical counties with different percentages of the Loess Plateau area based on geomorphic types, climate, water system, vegetation zone, and elevation, and analyzed the spatial and temporal differentiation characteristics of HQ and their influence mechanisms with the help of the InVEST model and geographical detector (GD) model. The results showed that: (1) From 2000 to 2020, cultivated land continued to decrease, while constructed land continued to increase. The three regions showed a decreasing trend of cultivated land and an increasing trend of forest as the area of the Loess Plateau within the region decreases. The three counties in the Transition Zone showed a decreasing trend of the increase in forest and an increasing trend of the increase in construction land as the area of the Loess Plateau within the county decreases. (2) From 2000 to 2020, HQ changes showed a bipolar sharpening phenomenon. The three regions showed a decreasing trend in moderately low grade and an increasing trend in moderately high grade as the area of the Loess Plateau within the region decreases. The three counties in the Transition Zone showed an increasing trend of the increase in low grade and a decreasing trend of the increase in moderately high grade as the area of the Loess Plateau within the county decreases. (3) From 2000 to 2020, the mean HQ showed a trend of first increasing then decreasing. HQ of the Loess Plateau, the Transition Zone, and the West Qinling Mountains showed a three‐level gradient of low, medium, and high. The Transition Zone generally showed the spatial distribution characteristics of low in the north and high in the south, and the low grade was mainly distributed in the Loess Plateau area within the county north. The formation of this geographical distribution verified that the ecological environment of the Loess Plateau had influenced the HQ of the West Qinling Mountains. (4) Land use intensity (LUI) and population density were the dominant factors causing spatial differentiation of HQ in the three regions, NDVI and NPP have always played a key role in the variation of HQ in the Transition Zone, and the synergistic enhancement effect between various factors promotes the change of regional HQ.

## Graphical abstract

The three regions showed a decreasing trend of cultivated land and an increasing trend of forest as the area of the Loess Plateau within the region decreases. From 2000 to 2020, HQ changes showed a bipolar sharpening phenomenon, and the three regions showed a decreasing trend in moderately low grade and an increasing trend in moderately high grade as the area of the Loess Plateau within the region decreases. Land use intensity and population density were the dominant factors causing spatial differentiation of HQ in the three regions, NDVI and NPP have always played a key role in the variation of HQ in the Transition Zone, and the synergistic enhancement effect between various factors promotes the change of regional HQ.

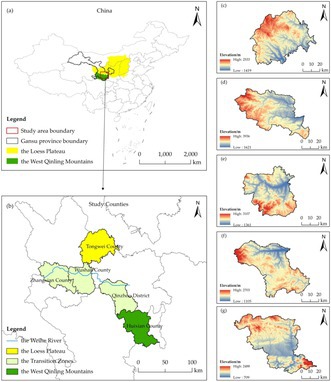

## 1 Introduction

Habitat quality (HQ) refers to the ability of a habitat to provide suitable conditions for the survival of an individual or population (Shi et al. 2024; Xie and Zhang 2023) and is an important indicator of measuring regional biodiversity level and ecosystem health (Zhang and Quan 2024; Wang and Cheng 2022), as well as a key indicator of reflecting the degree of coordination between socioeconomic development and the regional ecological environment (Zhang et al. 2024). Since the 21st century, with the acceleration of China's urbanization, the land use structure has been continuously transformed and reconstructed, leading to ecosystem service dysfunction and ecological environment fragmentation (Bai et al. 2019; Liu et al. 2024; Postek et al. 2019). Therefore, the evaluation of regional HQ based on land use patterns and the clarification of the spatial evolution patterns and influence mechanisms of HQ will help to implement habitat improvement measures accurately, which is of great significance to the rational planning of land resources and the realization of high‐quality development in the region.

There are two main methods to assess HQ; the first assessment method is based on field surveys to construct a habitat indicator system and is suitable for small‐scale and small river studies (Kong et al. 2022; Varol and Tokatlı 2021). This method has high requirements for data and is unable to carry out dynamic analyses of long time series (Xie and Zhang 2023). The second method is to use ecological models to assess HQ, such as the InVEST (Integrated Valuation of Ecosystem Services and Trade‐offs) model (Zhou et al. 2024; Zhu et al. 2022), the SolVES (Social Values for Ecosystem Services) model (Sherrouse et al. 2014; Zhang et al. 2021), and the HIS (the habitat suitability index) model (Bai et al. 2018; Duflot et al. 2018). Compared with other models, the InVEST model has been favored by various scholars because of its low data requirements, high confidence in the results, and strong spatial visualization (Li et al. 2021; Wang et al. 2024; Chen et al. 2023). At present, scholars have conducted a large number of basic and applied studies based on this model at different scales, such as revealing the spatial and temporal heterogeneity of HQ based on land use image data (Li et al. 2022; Mi et al. 2024), comparing the spatial expression of HQ in multiple scenarios (Tang et al. 2024; Li et al. 2024), exploring the regulatory mechanisms of urbanization on HQ (Song et al. 2020; Cheng et al. 2023), and clarifying the spatial and temporal correlation between landscape patterns and HQ (Yang et al. 2024; Gu et al. 2023). In general, the research on HQ has been relatively rich. However, a large number of studies have focused on the impacts of human development on HQ and the measures that can be taken, and there is a need for more in‐depth studies to comprehensively explore the mechanisms driving HQ. In existing research on factors affecting HQ, scholars have mostly explored the contribution of influencing factors to HQ based on models, of which the most common models are the geographical detector (GD) model (Yuan et al. 2023) and the geographically weighted regression (GWR) model (Cao et al. 2024). GD has been widely used because it can both quantify the degree of influence of a single factor on HQ and reflect the mechanism of influence on HQ when two factors interact (Xie and Zhang 2023; Chen et al. 2021; Jia et al. 2024). In addition, in the selection of the study area, most of the existing studies have taken nature reserves (Yang et al. 2021; Lin et al. 2024), basins (Tang et al. 2023; He et al. 2023), provinces, and municipalities (Luo et al. 2024; Xie et al. 2023) as a single study object, and few studies have been reported on the Transition Zone that is characterized by a clear geographical distribution. However, there are few studies that reveal the evolution trend of HQ from an ecological perspective by studying the Transition Zone with differences in geomorphic types.

The West Qinling Mountains belongs to the western extension of the Qinling Mountains, which is the most important ecological security barrier and “central water tower” in central China. Located between the Loess Plateau and the Sichuan Basin, the West Qinling Mountains is not only a part of China's north–south transition zone but also a transition zone between the Tibetan Plateau and the Qinba Mountains (Yang et al. 2013). It is of great significance for China's geographic structure, the formation of biodiversity patterns, and ecological security. However, under the stress of the Loess Plateau in the north, the northern part of the West Qinling Mountains has obvious transitional characteristics in terms of topography, climate, soil, vegetation, and other elements, which made HQ and its changes show specificity and territoriality (Chen et al. 2022). In recent years, with the acceleration of urbanization, the northern part of the West Qinling Mountains was facing difficulties such as imbalanced population and resource allocation, prominent contradictions in development and protection, and frustrated in high‐quality development, which had made the evolution trend of HQ more complex (Wang and Wang 2023). Therefore, selecting typical counties in the Loess Plateau, the Transition Zone, and the West Qinling Mountains to compare and analyze the spatial and temporal differentiation characteristics and explore the driving mechanism of HQ from an ecological perspective, which can not only reveal the ecological effects of the Loess Plateau on the HQ of the West Qinling, provide a practical guide to the land use planning and ecological construction of the West Qinling, but also expand the research idea of the assessment of HQ in the Transition Zone.

## 2 Materials and Methods

### 2.1 Study Area

**FIGURE 1:**
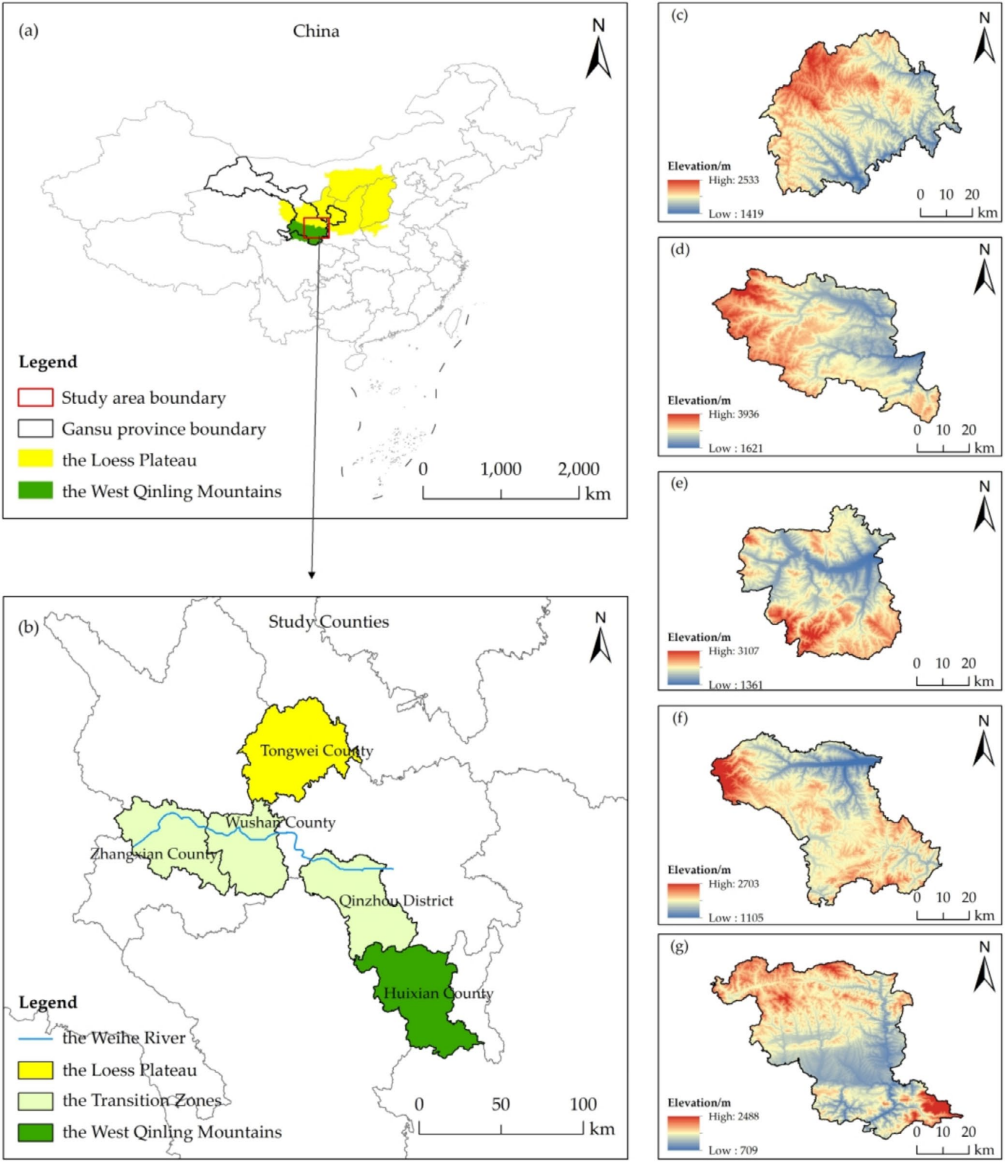
The research region's location, geomorphic types, and elevation (a) The Loess Plateau and the West Qinling Mountains' location in China, (b) the study counties' geomorphic types and specific location, (c) Tongwei County's elevation, (d) Zhangxian County's elevation, (e) Wushan County's elevation, (f) Qinzhou District's elevation, (g) Huixian County's elevation.

The West Qinling Mountains is located in the border region between southern Gansu Province and northern Sichuan Province, including 23 counties (districts) with a total area of about 84,109 km^2^ (Figure 1). This region has responded positively to China's policy of the Green for Grain Project due to the demand for ecological construction. The Green for Grain Project was based on the protection and improvement of the ecological environment, in which sloping farmland that is prone to soil erosion will be stopped from cultivation in a planned and systematic manner, and trees will be planted and forest cover restored in accordance with the principle of planting suitable trees on suitable land.

County (district) is one of the administrative units in China and plays an important role in regional socioeconomic development and ecological civilization construction. Therefore, it is important to explore the spatial and temporal variability of HQ at the county scale in order to accurately implement habitat improvement measures. Based on the above understanding, this study firstly identified the Transition Zone between the Loess Plateau and the West Qinling Mountains based on geographic location and climate types. Secondly, the study determined the distribution of geomorphic types within counties based on the boundaries of water systems (the northern part of the Weihe River Basin is the Loess Plateau, and the southern part is the Western Qinling Mountains). Lastly, we selected five study counties based on vegetation zone, mean elevation, and the area ratio of the Loess Plateau within the county (Table 1). By evaluating the changes in HQ in different time series, we attempted to clearly portray the ecological effects of the Loess Plateau on HQ of the West Qinling Mountains at the county scale.

**TABLE 1:** Study county selection criteria table.

Location	Climate	Water system	Geomorphic types	Vegetation zone	Mean elevation/m	Area ratio of the Loess Plateau	Study county
The Loess Plateau	Temperate semiarid monsoon climate	The Zuli River Basin	Loess hilly area	Typical grassland	1000–3000	100%	TWC
The Transition Zone	Temperate semihumid monsoon climate	The Weihe River Basin	Divided by the Weihe River Valley, the northern part is the loess hilly area, and the southern part is the Qinling mounts area	Typical grassland, forest grassland	2500–3000	33%	ZXC
				Forest grassland	2000–2500	30%	WSC
				Forest grassland, forest	1500–2000	10%	QZD
The West Qinling Mountains	Transition zone from subtropical to temperate	The Jialing River Basin	Qinling mounts area	forest	1000–1500	0%	HXC

Located in the core area of the Loess Plateau of central Gansu, Tongwei County (TWC) is a key county in the ecological function zone of soil and water conservation, and the terrain is high in the west and low in the east. The Transition Zone was divided into three levels based on altitude: 2500–3000 m, 2000–2500 m, and 1500–2000 m; Zhangxian County (ZXC), Wushan County (WSC), and Qinzhou District (QZD) were selected as representative counties based on vegetation zone and the proportion of the Loess Plateau area in the county. ZXC has the Loess Plateau area of 33%, with a terrain that is high in the west and low in the east. WSC has the Loess Plateau area of 30%, with a terrain that is high in the north and low in the south. QZD has the Loess Plateau area of 10%, with a terrain that is high in the west and low in the east. Huixian County (HXC) is located in the hinterland of the West Qinling Mountains and the Huicheng Basin in the upper reaches of the Jialing Basin, and the entire terrain makes a “concave” shape from north to south.

### 2.2 Data Sources

The land use/cover data (2000, 2020, 2020) obtained from the global 30 m land cover data (http://www.globallandcover.com), and we reclassified it into seven categories based on the land use characteristics of the study counties: cultivated land, forest, grassland, wetland, water, construction land, and unused land. Elevation, slope, and degree of topographic relief data were calculated from digital elevation model (DEM) data from the Geospatial Data Cloud Platform (http://www.gscloud.cn). Mean annual temperature and annual rainfall data were derived from the National Tibetan Plateau Science Data Centre (https://data.tpdc.ac.cn). Normalized difference vegetation index (NDVI), net primary production (NPP), soil type, soil erosion, gross domestic product (GDP), and nighttime light data were derived from the Resource and Environmental Sciences Data Centre of the Chinese Academy of Sciences (http://www.resdc.cn). Population density was derived from the World Population Grid data (https://hub.worldpop.org). Land use intensity (LUI) was calculated from land use data and land use intensity value. The data processing and image generation were achieved by ArcMap10.6, InVEST model, and Origin 2021. In this study, all data were resampled to a resolution of 30 m using the ArcMap10.6 platform, and the coordinate system was converted to WGS_1984_UTM_ Zone_48n.

### 2.3 Methods

#### 2.3.1 The Land Use Rate Model

The dynamic degree of land use can be used to describe changes in a certain land type of land use during the study period (Li et al. 2022) and is calculated using the following Formula (1):

(1) $$K=\frac{U_{b}-U_{a}}{U_{a}}\times \frac{1}{T}\times 100\%.$$

In the formula, *U*_*a*_ is the area of a land use type in the earlier period, *U*_*b*_ is the area of a land use type in the later period, and *T* is the interval between the two periods.

#### 2.3.2 Land Use Intensity

This study used land use intensity (LUI) to reflect the extent to which human activity interfered with land. Land use intensity value referenced the research result of Li et al. (2019), with a LUI of 0.2 for cultivated land, 0.03 for forest, 0.05 for grassland, 0.01 for wetland, water, 0.7 for construction land, and 0.01 for unused land. Based on the above data, LUI is calculated using the following Formula (2):

(2) $$\mathrm{LUI}=\sum_{i=1}^{n}\frac{S_{i}\;}{\sum_{i=1}^{n}S_{i}}\times D_{i}.$$

In the formula, *i* is land use type, *S*_*i*_ is land use intensity value of land use type *i*, and *D*_*i*_ is land use area of land use type *i*.

#### 2.3.3 InVEST Model

This study used the Habitat Quality module of InVEST 3.12.0 software to explore the spatial and temporal evolution of HQ in the study counties from 2000 to 2020. This model generates spatial maps of HQ based on land use data, distance of influence, and spatial weights of threat factors, habitat suitability of each land use type, and sensitivity of each habitat type to threat factors (Shi et al. 2024; Wang and Cheng 2022). The formula is as follows:

(3) $$D_{\mathrm{xj}}=\sum_{r=1}^{R}\sum_{y=1}^{Y_{r}}\;\left(W_{r}/\sum_{r=1}^{R}W_{r}\right)\;r_{y}i_{\mathrm{rxy}}\beta_{x}S_{\mathrm{jr},}$$

(4) $$\text{Linear decay}:i_{\mathrm{rxy}}=1-\left(\frac{d_{\mathrm{xy}}}{d_{\text{rmax}}}\right),$$

(5) $$\text{Exponential decay}:i_{\mathrm{rxy}}=\exp\;\left(\frac{-2.99d_{\mathrm{xy}}}{d_{\text{rmax}}}\right),$$

(6) $$Q_{\mathrm{xj}}=H_{j}\left[1-\left(\frac{D_{\mathrm{xj}}^{z}}{D_{\mathrm{xj}}^{z}+k^{z}}\right)\right].$$

In the formula, *D*_*xj*_ is the degree of habitat degradation of raster *x* in habitat type *j*; *R* is the number of threat factors; *Y*_*r*_ is the number of rasters of the threat source; *W*_*r*_ is the weight of the threat source *r* (0*–*1); *r*_*y*_ is the stress value of raster *y*; *i*_*rxy*_ is the threat level of *r*_*y*_ of raster y to raster *x*; *β*_*x*_ is the accessibility of the threat source to raster *x* (0 for legally protected areas and 1 for the rest of the area); *S*_*jr*_ is the sensitivity of the habitat type j to the threat source *r*; *d*_*xy*_ is the straight‐line distance of raster *x* from raster *y*; *d*_*rmax*_ is the maximum threat distance of threat source *r*; *Q*_*xj*_ is the HQ index for raster *x* in habitat type *j* (0*–*1); *H*_*j*_ is the habitat suitability of habitat type *j*; *k* is the half‐saturation constant, usually taken as half the degree of habitat degradation; *z* is the normalization constant (*z =* 2.5).

Considering the actual situation of the study county, cultivated land, construction land, and unused land were defined as threat sources; according to the InVEST model instruction manual and reading of similar papers (Wang and Cheng 2022; Zhu et al. 2022; He et al. 2023; Liang et al. 2022), the coercive distances and weights of the threat sources, the habitat suitability of the land use types, and the sensitivities of each habitat type to the threat factors were then determined (Table 2, Table 3).

**TABLE 2:** Habitat threat factors and threat degree.

Threat factors	Maximum effective distance (km)	Weight	Decay type
Cultivated land	1.5	0.3	linear
Construction land	6	0.6	exponential
Unused land	4	0.1	linear

**TABLE 3:** Habitat suitability and its sensitivity to threat factors.

Habitat type	Habitat suitability	Cultivated land	Construction land	Unused land
Cultivated land	0.4	0	0.4	0.4
Forest	1	0.8	0.6	0.6
Grassland	0.6	0.5	0.6	0.6
Wetland	1	0.7	0.8	0.5
Water	0.8	0.65	0.7	0.75
Construction land	0	0	0	0
Unused land	0.2	0	0	0

#### 2.3.4 Geographical Detector (GD) Model

GD is a statistical method used to detect spatial heterogeneity and reveal drivers (Wang et al. 2010). The core idea is based on the fact that if the independent variable has an effect on the dependent variable, then the spatial distributions of the independent and dependent variables are similar. One of the main advantages of GD is that it can detect both numerical and qualitative data, and can calculate and compare the explanatory power of each single factor and the explanatory power of the two factors under interaction, so as to clarify the degree of influence of the independent variable on the dependent variable and the relationship between them. The method consists of four main components: divergence and factor detection, interaction detection, risk detection, and ecology detection. In this study, we mainly used divergence and factor detection, and interaction detection in the GD to analyze the mechanism of the influence of drivers on the spatial heterogeneity of HQ.

Divergence and factor detection were used to measure the explanatory power of different drivers (*X*) on HQ (*Y*), which was expressed as q‐value (Xie and Zhang 2023; Wang et al. 2010), and were calculated by the following formula:

(7) $$q=1-\frac{\sum_{h=1}^{L}N_{h}\sigma_{h}^{2}}{N\sigma^{2}}.$$

In the formula, *q* is the explanatory power of the different factors for HQ; *h* is the classification or partitioning of the factor; *N*_*h*_ and *N* are the number of cells in class *h* and the whole region; *σ*_*h*_^2^ and *σ* ^2^ are the *Y* value variances of class *h* and the whole region; *Nσ* ^2^ is the total variance of the whole region.

Interaction detection was used to measure the explanatory power of the two factors on HQ under interaction (Xie and Zhang 2023; Wang et al. 2010). The relationship between the two factors is shown in Table 4.

**TABLE 4:** Types of two‐factor interaction results.

Judgment basis	Interaction type
$q\left(X1\cap X2\right)<\mathrm{Min}\left(q\left(X1\right),q\left(X2\right)\right)$	Nonlinear attenuation
$\mathrm{Min}\left(q\left(X1\right),q\left(X2\right)\right)<q\left(X1\cap X2\right)<\mathrm{Max}\left(q\left(X1\right),q\left(X2\right)\right)$	Single‐factor nonlinear attenuation
$q\left(X1\cap X2\right)>\mathrm{Max}\left(q\left(X1\right),q\left(X2\right)\right)$	Double‐factor enhancement
$q\left(X1\cap X2\right)=q\left(X1\right)+q\left(X2\right)$	Independence
$q\left(X1\cap X2\right)>q\left(X1\right)+q\left(X2\right)$	Nonlinear enhancement

HQ spatial heterogeneity is the result of the combined effect of many factors (Bai et al. 2019), such as ecological factors that provide the developmental context of the natural environment, determining the initial spatial distribution pattern of HQ; human factors that measure the regional economic status and development level, reflecting the degree of human interference with the habitat environment (Chen et al. 2021; Chen and Liu 2024). In order to identify the main driving factors the HQ of the study region, this study selects 13 indicators (Table 5) based on natural characteristics and socioeconomic development characteristics.

**TABLE 5:** Driving factors of HQ change.

Type	Driving factors		Data source
Ecological factors	Topography	Elevation *X*1 Slope *X*2 Degree of topographic relief *X*3	http://www.gscloud.cn Using Slope tool to calculate in ArcGIS based on DEM data Using Focal Statistics to calculate in ArcGIS based on DEM data
	Climate	Annual average temperature *X*4 Annual precipitation *X*5	https://data.tpdc.ac.cn https://data.tpdc.ac.cn
	Vegetation	NDVI index *X*6 NPP index *X*7	http://www.resdc.cn http://www.resdc.cn
	Soil	Soil type *X*8 Soil erosion *X*9	http://www.resdc.cn http://www.resdc.cn
Human factors	Economic level	GDP *X*10	http://www.resdc.cn
	Carrying capacity	Population density *X*11	https://hub.worldpop.org
	Social activity	Night time light data *X*12	http://www.resdc.cn
	Land use	Land use intensity *X*13	Using Formula (2) to calculate based on land use data

In ArcGIS 10.6, firstly, the factors were rasterized, including projection, resample, extraction, and calculation. Secondly, each factor was discretized and classified by using the quantile method, and as independent variables (*Xi*), HQ as the dependent variable (*Y*). Thirdly, based on studies at the county grid scale (Yang 2021), the fishing net creating tool was used to generate a 2200 m × 2200 m grid as a sampling point, the classified values of *Xi* and *Y* were extracted on the sampling points, and then, the generated data table was imported into the GeoDetector_2018 model to calculate the relevant results.

## 3 Results

### 3.1 Land Use Change Analysis

#### 3.1.1 Characteristics of Temporal Change in Land Use

**FIGURE 2:**
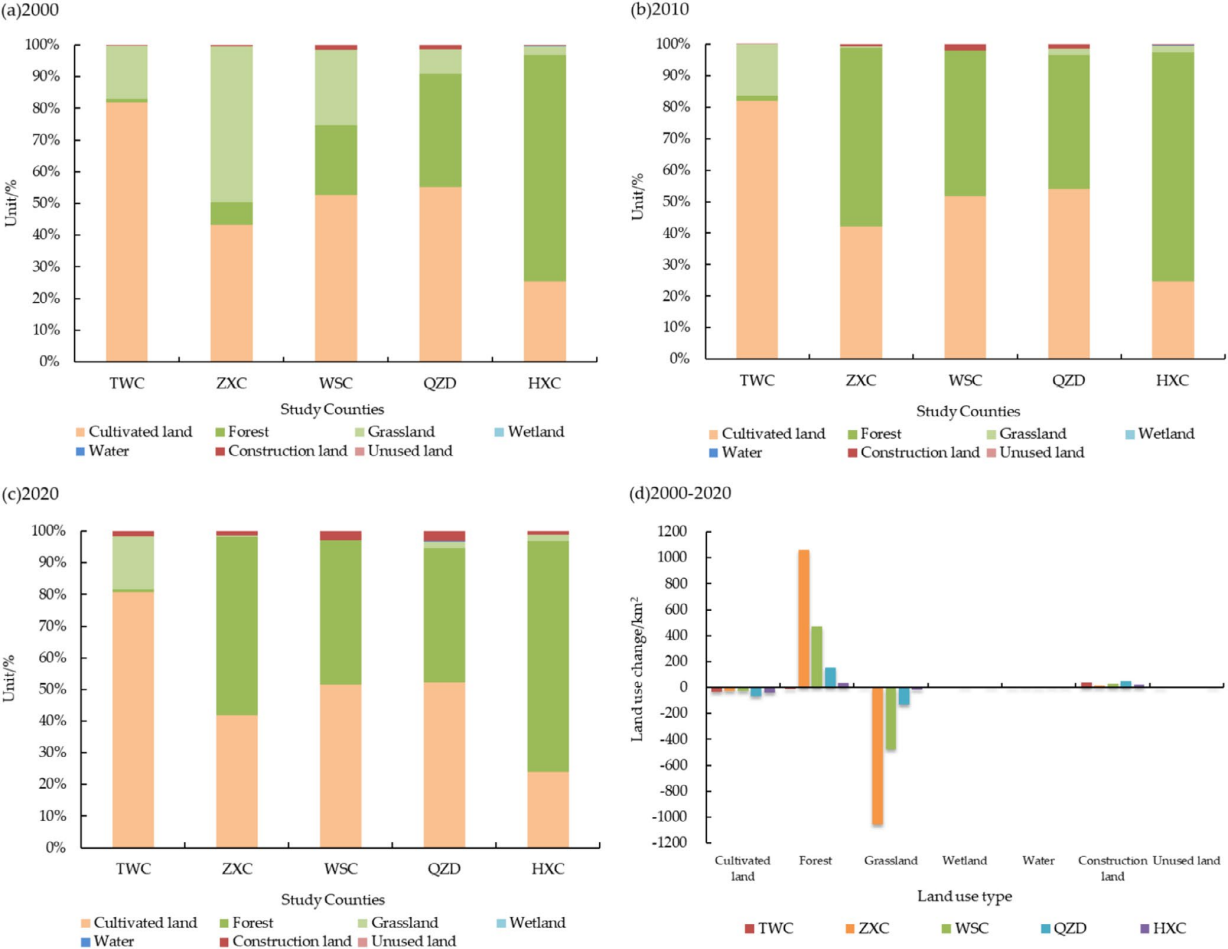
Proportion and change of land use types area in the study counties. (a) Area proportion of land use types in 2000, (b) Area proportion of land use types in 2010, (c) Area proportion of land use types in 2020, (d) Area change of land use types from 2000 to 2020.

The land use change in the study counties for the period 2000–2020 is shown in Figure 2; cultivated land, forest, grassland, and construction land were the main land use change types. From the analysis of land use area changes, construction land areas of the Loess Plateau (TWC) increased by 39.38 km^2^, while cultivated land and forest areas decreased by 35.44 and 10.63 km^2^, respectively. In the Transition Zone, forest and construction land areas increased, while cultivated land and grassland areas decreased during the study period. Forest areas of ZXC, WSX, and QZD increased by 1063.10, 472.15, and 153.07 km^2^, respectively, followed by construction land and grassland areas; construction land of QZD increased by 47.63 km^2^, and grassland of ZXC decreased by 1056.06 km^2^. The land use area change of the West Qinling Mountains (HXC) was mainly reflected in forest, construction land, and cultivated land. Forest and construction land increased by 33.33 and 20.89 km^2^, respectively, and cultivated land decreased by137.89 km^2^.

From the analysis of land use dynamics (Table 6), the land use dynamics of cultivated land were all negative in study counties, belonging to the type of land use that was continuously decreasing, and cultivated land in QZD had the most significant decrease; the land use dynamics of construction land were all positive, and the latter 10 years had a greater degree of increase than the first 10 years, and the land use of TWC from 2010 to 2020 reached 114.42%. Forests showed a dynamic process of increasing then decreasing, but the rest of the counties except TWC increased as a whole. Grassland except TWC showed a dynamic process of continuous decrease, and the area of grassland decreased with the decrease of the Loess Plateau area within the county.

**TABLE 6:** Land use dynamics from 2000 to 2020.

Land use type	2000–2010 land use dynamics/%					2010–2020 land use dynamics/%				
	TWC	ZXC	WSC	QZD	HXC	TWC	ZXC	WSC	QZD	HXC
Cultivated land	−0.002	−0.20	−0.21	−0.20	−0.25	−0.15	−0.08	−0.04	−0.32	−0.31
Forest	3.84	67.60	11.10	1.85	0.17	−5.07	−0.02	−0.12	−0.04	0.01
Grassland	−0.27	−9.92	−10.00	−7.19	−2.36	0.43	−3.47	−2.20	−0.08	−0.15
Wetland	0.00	0.00	3.46	0.00	40.73	0.00	0.00	0.16	0.00	−7.60
Water	−6.48	15.22	−1.72	−2.98	2.84	−7.82	−2.76	−0.33	5.28	0.21
Construction land	0.55	5.39	4.04	0.94	0.12	114.42	7.98	3.74	13.95	27.82
Unused land	−2.00	0.00	0.00	0.00	0.00	0.00	0.00	0.00	0.00	0.03

Overall, due to the differences in geomorphic types within the county, the trends in area change and the extent of change are different for each land use type. In terms of geographic location, the three major regions (the Loess Plateau, the Transition Zone, the West Qinling Mountains) showed a decrease in cultivated land and an increase in forest as the area of the Loess Plateau decreased. For the three counties in the Transition Zone, from 2000 to 2020, the decrease in cultivated land increased with the decrease in the area of the Loess Plateau within the county, the increase in forest decreased with the decrease in the area of the Loess Plateau within the county, the decrease in grassland decreased with the decrease in the area of the Loess Plateau within the county, and the increase in construction land increased with the decrease in the area of the Loess Plateau within the county.

#### 3.1.2 Characteristics of Spatial Distribution in Land Use

**FIGURE 3:**
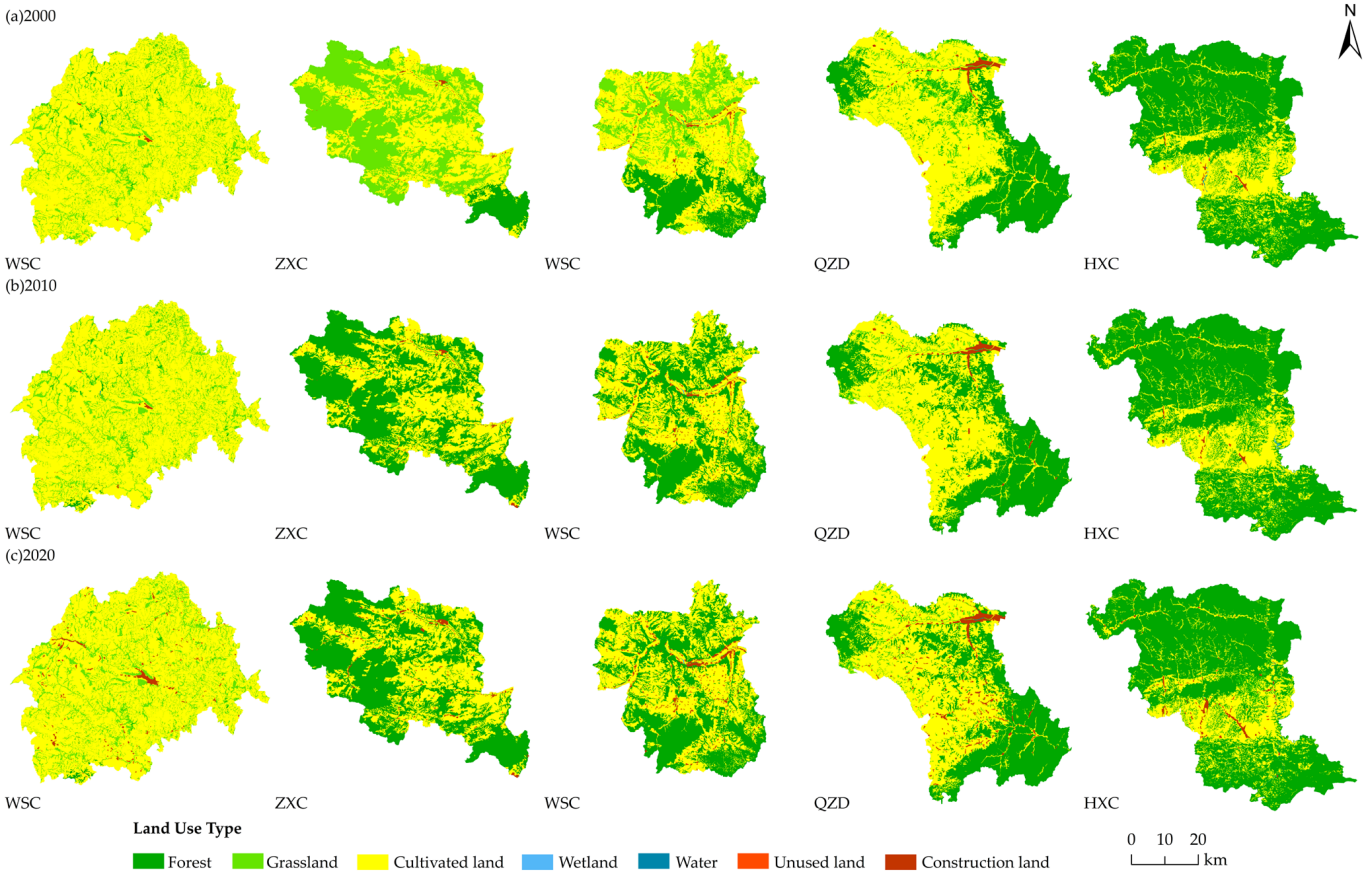
Spatial distribution maps of land use change in the study counties. (a) Spatial distribution of land use types in 2000, (b) Spatial distribution of land use types in 2010, (c) Spatial distribution of land use types in 2020.

The spatial distribution of land use types in the study counties for the period 2000–2020 is shown in Figure 3. There were significant differences in the spatial distribution of land use types across geomorphic features. Land use types of the Loess Plateau (TWC) were dominated by cultivated land, amounting to more than 80% of the total area, and were scattered throughout the county, followed by grassland. The proportion of construction land was relatively small, mainly distributed in the central region, and showed a spreading trend toward the surrounding areas over time. The land use types of the Transition Zone (ZXC, WSC, QZD) were dominated by cultivated land and forest; forest was mainly distributed in the West Qinling Mountains area in the southern part of the counties, and construction land was mainly distributed in the Loess Plateau area in the northern part of the counties. HXC, in the West Qinling Mountains, had over 70% of its land area in forest, while the proportion of cultivated land was maintained at around 24%. Forest was mainly distributed in the Qinling Mountains areas in the north and south of the county, while cultivated land and construction land were mainly distributed in the river valley area in the center.

### 3.2 Temporal and Spatial Variation Characteristics of HQ

#### 3.2.1 Characteristics of Temporal Change in HQ

In this study, the InVEST model was used to assess the HQ, which ranged from 0 to 1, with the closer to 1, the better the HQ condition (Wang and Cheng 2022; Zhu et al. 2022). In order to analyze in‐depth the impacts of land use change on HQ, using the Re‐classify tool of the ArcGIS 10.6 software platform, HQ was divided into five grades: 0–0.2, 0.2–0.4, 0.4–0.6, 0.6–0.8, 0.8–1, they, respectively, correspond to the five grades of low, moderately low, medium, moderately high, and high.

**FIGURE 4:**
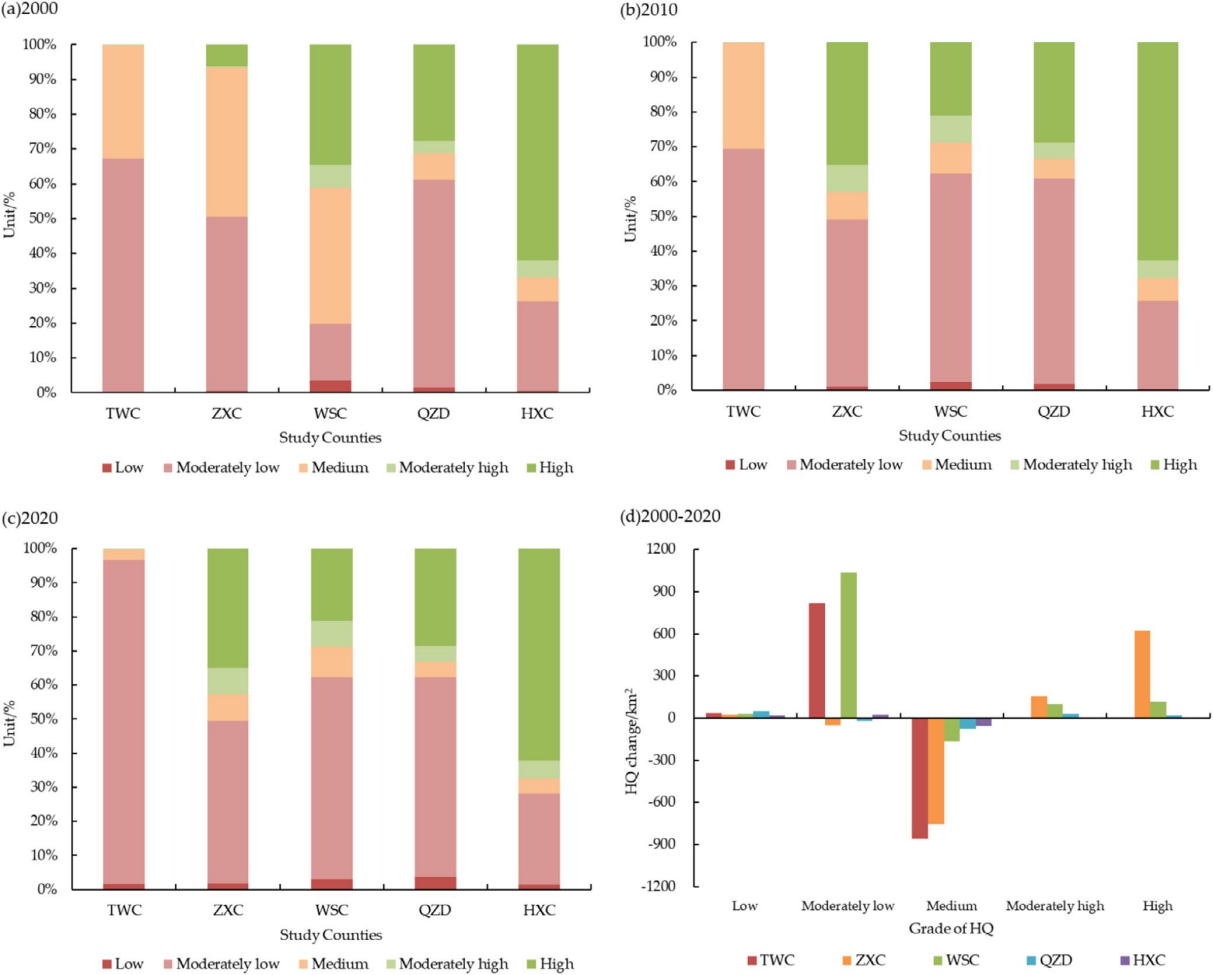
Temporal change of the proportion of HQ grade, (a) Area proportion of HQ grade in 2000, (b) Area proportion of HQ grade in 2010, (c) Area proportion of HQ grade in 2020, (d) Area change of HQ grade from 2000 to 2020.

The HQ change in the study counties for the period 2000–2020 is shown in Figure 4; moderately low grade and medium grade were the main HQ change types. HQ of the Loess Plateau (TWC) was dominated by moderately low and medium grades, with the sum of the areas of the two accounting for nearly 99% of the total, of which the area ratio of moderately low grade was more than 65%. The area of the medium grade decreased by 858.19 km^2^, the remaining four grades showed an increasing state, and the area of the lower grade increased by 820.76 km^2^. For the Transition Zone, the sum of moderately low grade and medium grade areas exceeded 50%, and the decrease in medium grade area showed a decreasing trend as the area of the Loess Plateau within the county decreased. In ZXC, the area ratio of medium grade decreased by 34.78%, the area ratios of low and high grade increased by 1.17% and 28.75%, respectively, and the area ratio of high grade increased by 28.75%. In WSC, the area ratio of medium grade decreased by 30.16%, and moderately low grade and moderately high grade increased by 43.01% and 1.29%, respectively. In QZD, the area ratio of medium grade decreased by 3.24%, and the area ratios of moderately low grade and moderately high grade increased by 2.16% and 1.17%, respectively. HQ of the West Qinling Mountains (HXC) was dominated by the high grade, and the area ratio was maintained at about 62%. The changes in the area ratio of each class were small, but the area ratio of the medium grade decreased by 2.10%, and the area ratios of the remaining four grades showed a slight increase.

Overall, the degree of change in the various grades of HQ varied across the regions, but all showed a two‐tiered sharpening phenomenon, with a decrease in the area of the medium grade and an increase in the low grade and high grade. For the Transition Zone, with the area of the Loess Plateau decreasing within the county, the amount of increase in the low grade showed an expanding state, the amount of decrease in the medium grade showed a declining state, and the amount of increase in both the high grade and moderately high grade showed a declining state.

#### 3.2.2 Characteristics of Spatial Change in HQ

**FIGURE 5:**
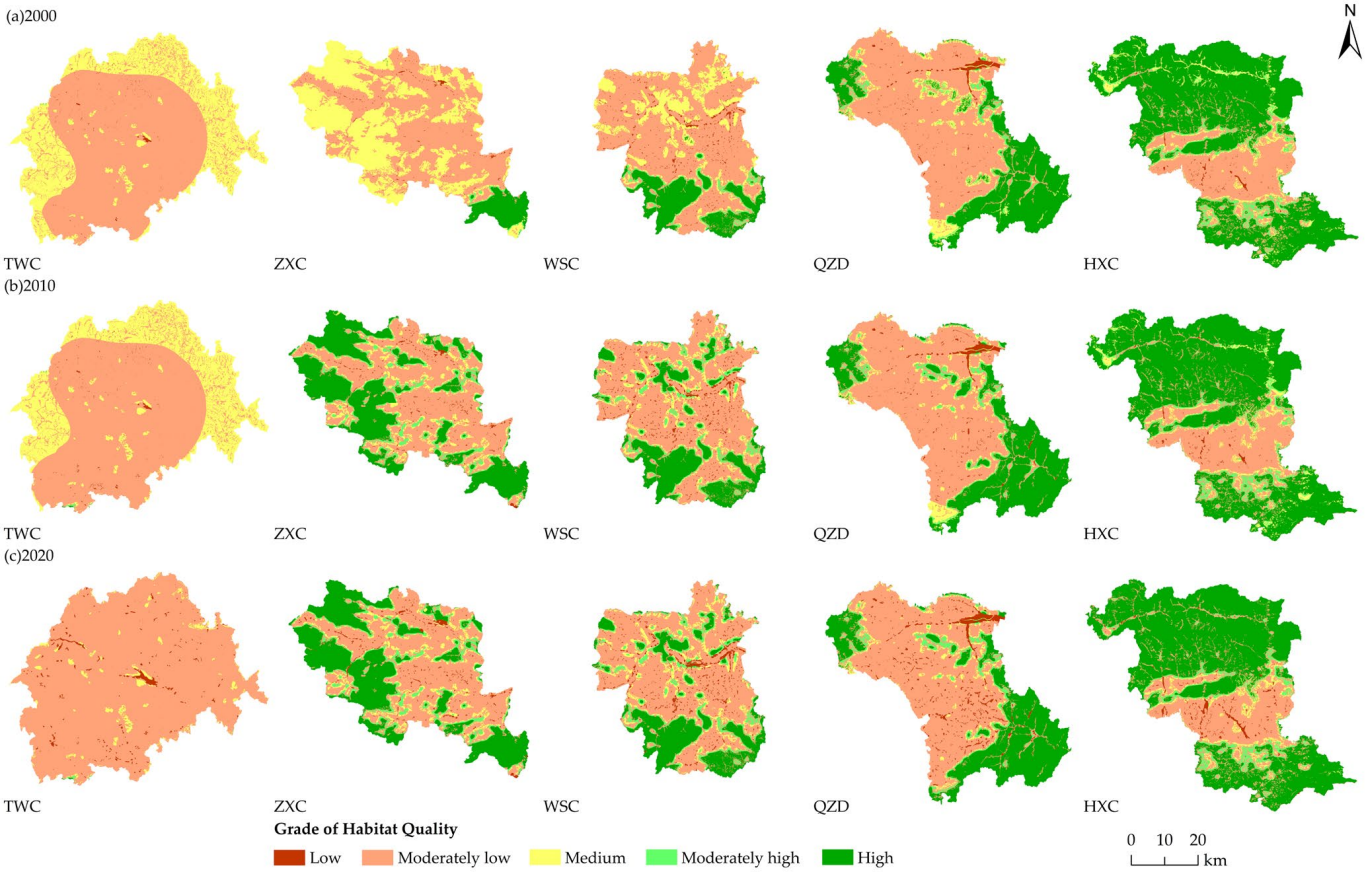
Spatial distribution maps of HQ in the study counties. (a) Spatial distribution of HQ in 2000, (b) Spatial distribution of HQ in 2010, (c) Spatial distribution of HQ in 2020.

In terms of spatial distribution (Figure 5), there was a clear spatial distribution of HQ in the study counties, and it was broadly consistent with the distribution of land use types. HQ was high in areas where forest was distributed and low in areas where construction land was distributed. The Loess Plateau (TWC) had a wide distribution of moderately low habitat, with land types dominated by cultivated land and grassland; high and moderately high habitats were mainly located in the southern region with land types dominated by forest; and medium and low habitats were sporadically distributed with land types dominated by grassland and construction land.

From the Transition Zone, moderately low and high habitats were the dominant distribution types, and moderately low habitat was widely distributed throughout the county, while high habitat was mainly found in the West Qinling Mountains within the county. Low habitat was mainly found in the river valley area in the central part of the county and in the Loess Plateau area in the north, with cultivated land and construction land as the main land types. High habitat was mainly distributed in the West Qinling Mountains in the southern part of the county, with forest as the main land type. Moderately high and medium habitats were distributed in strips along the high habitat in a sequential outward direction.

HQ of the West Qinling Mountains (HXC) showed an overall distribution pattern of high in the north and south, and low in the middle. HQ was dominated by high habitat and was distributed in the northern and southern parts of the county, with forest as the main land use type. Low and moderately low habitats were mainly distributed in the central region, where there was a large amount of cultivated land and construction land, which had been subjected to large amounts of human interference, resulting in a serious disruption of the HQ. Moderately high and medium habitats were distributed in the vicinity of the high habitat and were in the form of a band in a sequential manner outward.

### 3.3 Gradient of Change in HQ With Geomorphic Types

From 2000 to 2020, mean HQ all showed a first increasing then decreasing trend in the time series (Table 7); this was closely related to the full implementation of the national policy of the Green for Grain Project in 2002. After entering 2011, the slowing down of the Green for Grain Project and the sprinting phase of the Development of the Western Region in China led to a slight decline in HQ of the study counties in the period 2011–2020.

**TABLE 7:** Mean habitat index change in the study counties.

Year	TWC	ZXC	WSC	QZD	HXC
2000–2010 change	0.0002	0.1266	0.0322	0.0060	0.0046
2000–2010 Rate of change/%	0.05%	25.52%	6.45%	1.06%	0.60%
2010–2020 change	−0.0035	−0.0043	−0.0023	−0.0098	−0.0025
2010–2020 Rate of change/%	−0.91%	−0.69%	−0.43%	−1.72%	−0.92%

**FIGURE 6:**
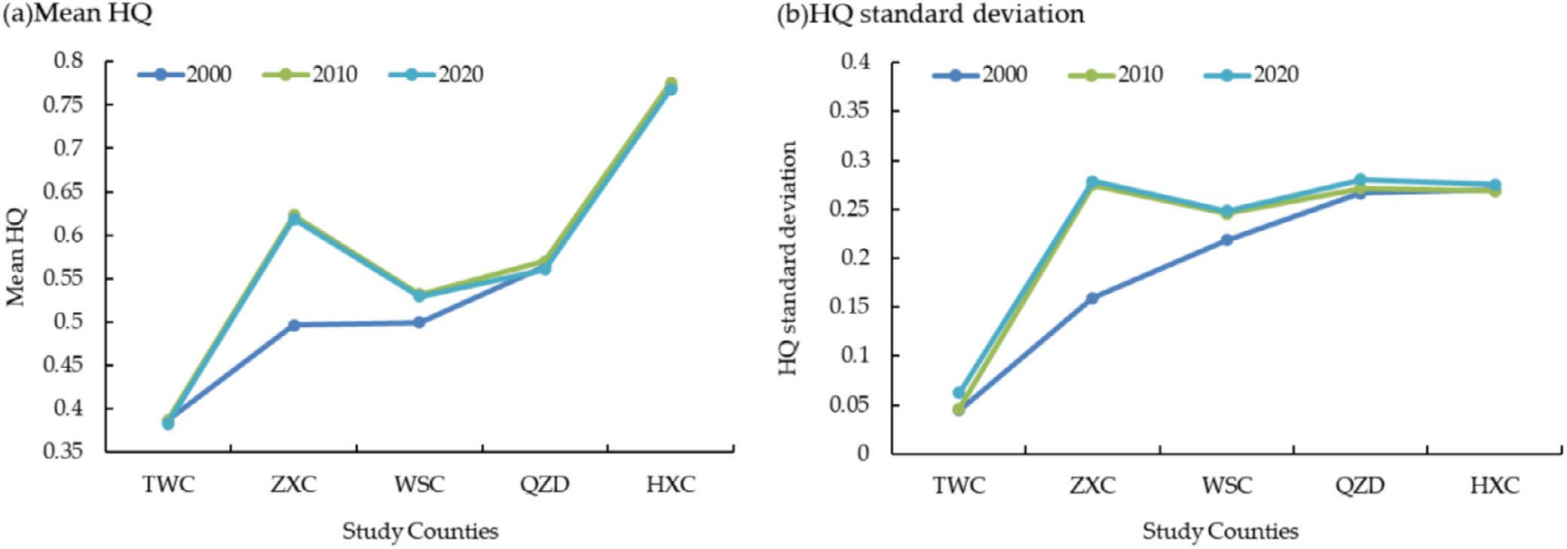
Temporal change of HQ in the study counties. (a) Changes of mean HQ from 2000 to 2020. (b) Changes of HQ standard deviation from 2000 to 2020.

With changes in geospatial scales, mean HQ of the Loess Plateau (TWC), the Transition Zone (ZXC, WSC, QZD), and the West Qinling Mountains (HXC) showed a three‐level gradient features of low, medium, and high (Figure 6a). From 2000 to 2020, HQ of the Loess Plateau region (TWC) maintained a low grade, decreased by 0.0033, while the Transition Zone (ZXC, WSC, QZD) had a moderate grade of HQ, with an enhancement rate of 9.51%, HQ of the West Qinling Mountains (HXC) maintained a high grade and declined by 0.0025. The formation and changes in this geographical distribution conform to the natural conditions and economic development levels of different territories. In terms of changes in the HQ standard deviation (Figure 6b), the standard deviation of HQ in the study counties all showed an increasing trend, which indicated that spatial differences in HQ had been gradually increased over the 20‐year period. HQ standard deviation also showed variability across geomorphic types, which increased as the area of the Loess Plateau within the county decreases. The formation of this geographic distribution further validated that the ecological effects of the Loess Plateau influence HQ of the West Qinling Mountains.

### 3.4 Driving Factors of HQ Change

#### 3.4.1 Divergence and Factor Detection

The geographical detector model was used to detect the degree of influence of different factors on the spatial distribution of HQ, with an explanatory power q ranging from 0 to 1 (Wang et al. 2010). The larger the value of q, the stronger the explanatory power of independent variables (*Xi*) on the dependent variable (*Y*), the greater the degree of influence on the spatial distribution of HQ.

**FIGURE 7:**
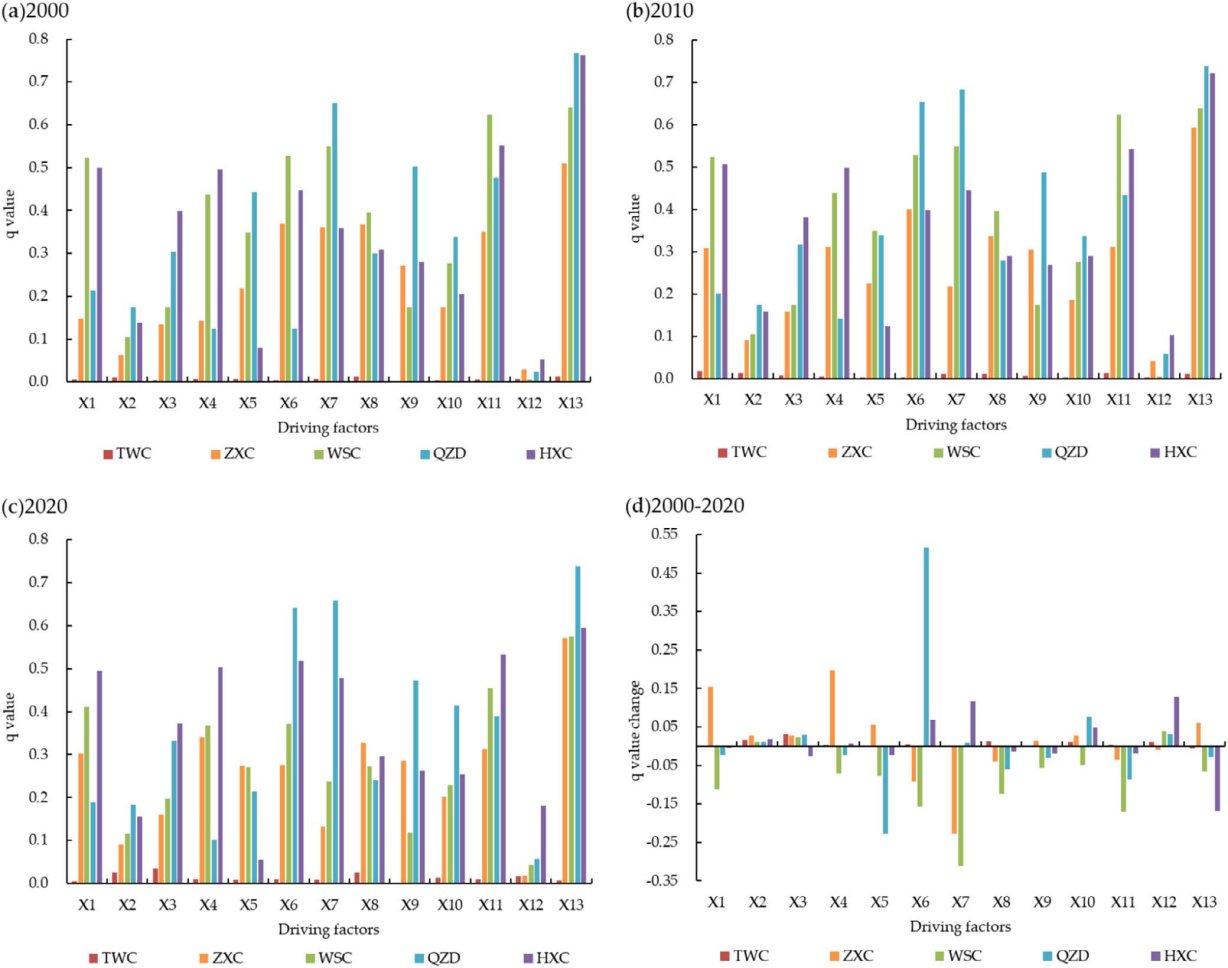
Detection results of HQ impact factors, (a) The q value of HQ impact factors in 2000, (b) The q value of HQ impact factors in 2010, (c) The q value of HQ impact factors in 2020, (d) The q value change of HQ impact factors from 2000 to 2020.

Based on the results of the detection of all variables in the three zones from 2000 to 2020, it was found that each factor played a different level of role in the change of HQ (Figure 7). The Loess Plateau (TWC) had severe soil erosion and a fragile natural environment. Since 2000, HQ had decreased and spatial differences had continued to increase. The key factors that affect the spatial differences in HQ were mainly topography factor, soil factor, and LUI. In the past 20 years, the influence of temperature, precipitation, and NDVI of Ecological factors had increased to varying degrees, indicating the important impact of climate factors and vegetation cover on the HQ of the Loess Plateau, which was closely related to the poor natural conditions of the Loess Plateau. The q‐values of LUI and population density of human factors were relatively high in all three time periods, indicating the significant impact of land use changes caused by human activities on HQ. HQ in the Transition Zone (ZXC, WSC, QZD) had been improved, and factor detection in three periods showed that LUI, population density, NDVI, and NPP were the main factors. The q‐values of NPP and population density showed a decreasing trend, indicating that changes in vegetation factors and population density had a significant impact on HQ. Over the past 20 years, HQ in the West Qinling Mountains (HXC) had first increased then decreased, with an overall decrease of 0.0025. The q‐values of LUI, population density, elevation, and temperature in the three periods remained stable at around 0.5, and the q‐values of NDVI and NPP showed an increasing trend, indicating that vegetation factor was the main factor affecting the changes in HQ of the West Qinling Mountains.

#### 3.4.2 Interaction Detection

**FIGURE 8:**
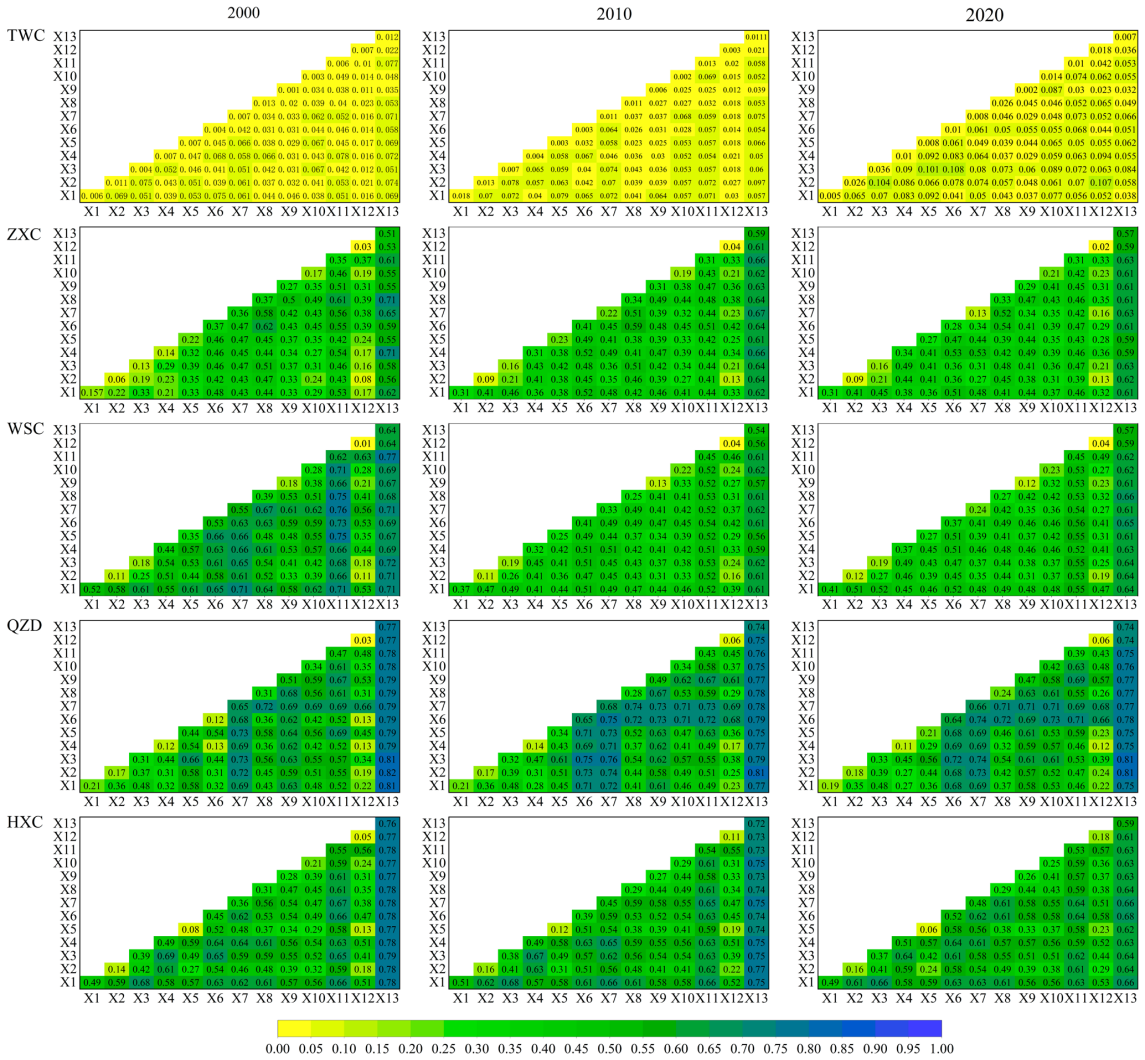
Interaction detection results of HQ impact factors.

The results of the two‐factor interaction test are shown in Figure 8. Overall, the interactions between the factors showed a synergistic enhancement during the period 2000–2020, and the interactions between the ecological and human factors were stronger than the interactions within the ecological factors and the interactions within the human factors.

For TWC located in the Loess Plateau, the terrain factor interacted most strongly with other factors, with a q‐mean value of 0.056, and the q‐value tended to increase with the change of time, while LUI interacted with other factors to a slightly lesser extent, with the mean q‐value of 0.053. Analyzed in terms of the degree of temporal change, NDVI and temperature factors interacted with other factors to the greatest extent, with the mean q‐value increasing by 0.018 and 0.017. For the three counties located in the Transition Zone (ZXC, WSC, QZD), LUI showed a strong interaction with all other factors, with the mean q‐value maintained above 59%, followed by the population density interacting with other factors, with the mean q‐value of Zhang County, Wushan County, and Qinzhou District of 0.46, 0.58, and 0.58, respectively. Thirdly, the vegetation factor interacted with other factors; the mean q‐value of the three counties was 0.44, 0.52, and 0.66, respectively. From 2000 to 2020, the vegetation factor interacting with other factors had the greatest change, in which ZXC and WSC showed a decreasing trend and QZD showed an increasing trend, which was opposite to the trend of the change of HQ, suggesting that the vegetation factor was the main factor influencing the change of the regional HQ. For HXC located in the West Qinling Mountains, the interaction between LUI and other factors was the strongest, with the mean q‐value of 0.718, and the population density and the elevation factors had the second strongest interaction with other factors, with the mean q‐value of 0.615 and 0.601, respectively. Analyzed from the perspective of temporal change, the interactions between NDVI and NPP and other factors showed a trend of enhancement, which indicated that changes in HQ of HXC were significantly influenced by vegetation factors significantly.

## 4 Discussion

### 4.1 Temporal and Spatial Variation of HQ

Geomorphology is the result of the combined effect of internal and external geological forces on the earth's crust and is the basic environmental condition that influences the formation of the biological environment and constrains human activities (Wang and Cheng 2022). Based on the distribution of geomorphic types, this study comparatively analyzed the spatial and temporal differences of HQ in the three regions (the Loess Plateau, the Transitional Zone, the West Qinling Mountains) and clarified the ecological effect of the Loess Plateau on HQ of the West Qinling Mountains, which can provide important theoretical references for coordinated regional development.

With the change of time scale, HQ of the three regions showed a bipolar sharpening phenomenon, which was manifested by the decrease of medium‐quality area and the increase of low‐ and high‐quality areas. This result was similar to the findings of Xie et al. (2023) and Yang (2021), Xie et al., who found that the habitat area of the median‐grade drastically dropped, but the habitat areas of the low‐grade and the high‐grade in habitat simultaneously expanded in their study of the dynamics of HQ in urban areas of China; Yang et al., who found that the low‐quality habitats continued to increase, while the medium‐quality habitats and the higher‐quality habitats continued to decrease, and the high‐quality habitats showed an increasing trend in their study on the spatial and temporal evolution of HQ in mountainous areas of China. This study result was closely related to regional economic development and ecological protection. The promotion of urbanization and industrialization had led to an increase in demand for construction land, which inevitably encroached on surrounding ecological land, resulted in a decrease in cultivated land, grassland, and water area in the region, and thus expanded the low value areas of HQ. The implementation of the Green for Grain Project and the Land Reclamation Project had increased vegetation coverage and effectively improved regional HQ (Gao et al. 2022). Land use patterns directly determined the spatial distribution pattern of HQ (Li et al. 2022). With the change of geomorphic scales, there were significant differences in land use patterns among the Loess Plateau, the Transition Zone, and the West Qinling Mountains. The ratio of the sum of cultivated land and grassland area of the Loess Plateau (TWC) was close to 98%, and the ratio of forested area was only about 1%; the ratio of cultivated land and forested area of the three counties of the Transitional Zone was close to 1:1, and the ratio of forested area of the West Qinling Mountains (HXC) was more than 70%, and these land use patterns directly led to the three‐level gradient characteristic of HQ in the order of low, medium, and high. HQ in the Transition Zone generally showed a distribution pattern of low in the north and high in the south, and low habitat was mainly distributed in the Loess Hills area in the north, while high habitat was mainly distributed in the West Qinling Mountains in the southern part, where HQ of the mountains was greater than that of the loess hills. This result was similar to the findings of Zhang et al. (2022), who found that the spatial distribution of HQ and topography distribution patterns had correlation in their study of spatiotemporal variation of HQ in Liulin County of China, and concluded a relationship that HQ in mountainous area was greater than that in hilly area. Due to the higher altitude of the West Qinling Mountains, human activities were limited, vegetation cover was high, and HQ was good. The Loess Hills in the north, where a large amount of cultivated land and construction land was distributed, was a major population concentration area and economic development area, and human activities seriously threatened the surrounding environment, resulting in poorer HQ conditions.

### 4.2 Driving Mechanism of HQ

Analyzing the driving mechanism of HQ is essential for understanding the causes of HQ spatial variation and implementing habitat improvement measures (Xie and Zhang 2023). Based on the spatial differentiation of regional HQ, this study selected 13 factors from ecological attributes and human activity and quantified their influences with the help of the GD model.

The results of the GD model detection showed that LUI was the main factor in the formation of the spatial distribution pattern of HQ in the three regions. This result had similarity with the findings of Chen et al. (Chen et al. 2024), who found that the escalation in LUI was the main catalyst for the decline of HQ in their study of the influence of land use change on HQ in coal mining subsidence areas. Land is the material basis of human activities, and its use changes directly represent the use and transformation of natural ecosystems by human activities (Zheng and Li 2022). Cultivated land and construction land were frequently disturbed by human beings and have a high LUI value, thus showed a significant negative effect on HQ, while forest land and water had less human ecological intrusion and LUI was of low value, so showed a significant positive effect on HQ. There was a significant difference in regional LUI with changes in geomorphic scales. The three regions showed a gradual decrease in LUI with the decrease in the area of the Loess Plateau, and with the change in time scale, LUI showed a trend of first decreasing then increasing, which was opposite to the spatial change trend of HQ. Therefore, LUI showed a negative correlation with HQ; this result was similar to the findings of Liang et al. (2022), who found that urban expansion had a very serious negative impact on HQ in studying the relationship between urban expansion and HQ in the Loess Plateau of China. Enhanced LUI will expand the area of threat sources, leading to habitat degradation and HQ decline in the adjacent areas.

For the three counties in the Transition Zone, the vegetation factors also played a key role. As the area of the Loess Plateau decreases, the explanatory power of NDVI and NPP on HQ gradually became larger, indicating that the effect of the Loess Plateau on the HQ of the West Qinling Mountains was mainly reflected in the vegetation cover. NDVI and NPP are important parameters reflecting the growth status of vegetation, which were found to be mainly influenced by human activities and temperature (Hou et al. 2022). The three counties in the transition zone were all in the temperate semihumid monsoon climate zone, so the differences in vegetation cover among the three counties were mainly affected by human activities. The construction land transformed by human activities was mainly distributed in the Loess Plateau area in the northern part of the county, resulting in a decrease in vegetation coverage in the northern part of the county, and over time, the construction land was spreading outward in a radial manner. Therefore, when optimizing regional HQ in the future, we need to develop appropriate control measures based on the distribution characteristics of geomorphic types and economic development needs, optimize the layout of urban construction land, solve the development dilemma of low value HQ areas, coordinate the relationship between development and protection, and achieve sustainable development of the West Qinling Mountains.

### 4.3 Limitations of the Study

This study used the InVEST‐HQ model to explore the spatial distribution of HQ under different geomorphic types, and used the GD model to reveal the reasons for the spatial differentiation of HQ, intuitively reflecting the changing trend of HQ with the change of geomorphic distribution area. This could provide some inspiration for subsequent scholars to study the HQ of similar areas. However, some of the parameters required for the InVEST‐HQ model come from literature and expert experience, which made the evaluation results somewhat subjective. In the future, it is necessary to strengthen on‐site investigations to obtain more accurate threat source parameters and thus obtain more accurate evaluation results. The spatial differences of HQ are the result of a combination of multiple factors. Due to the availability of data, this study only selected 13 factors from nine aspects of soil, topography, vegetation, climate, economic development level, social carrying capacity, human activities, and land use. In the future, it is necessary to consider the impact of policy implementation and species invasion on HQ. In addition, when exploring the spatial differences of HQ at the geomorphic scale, this study only selected five typical counties for comparative analysis based on geomorphic attributes, climate, water system, vegetation zone, and altitude. In the future, it is necessary to expand the research scale and further clarify the ecological effects of geomorphic types.

## 5 Conclusions

In this study, we based our analysis on the global land cover data GlobeLand30 to analyze the characteristics of land use change and the spatial and temporal evolution of HQ with the help of land use dynamics and the InVEST model, and used the GD model to explore the driving mechanisms of the spatial differentiation of HQ. The main conclusions are as follows:

1. From 2000 to 2020, the types of land use changes in the study counties were mainly dominated by cultivated land, forest, grassland, and construction land. Cultivated land continued to decrease, while construction land continued to increase. The three regions (the Loess Plateau, the Transition Zone, the West Qinling Mountains) showed a decreasing trend of cultivated land and an increasing trend of forest as the area of the Loess Plateau within the region decreases. The three counties in the Transition Zone (ZXC, WSC, QZD) showed a decreasing trend of the increase in forest and an increasing trend of the increase in construction land as the area of the Loess Plateau within the county decreases.
2. From 2000 to 2020, HQ changes in the study counties showed a bipolar sharpening phenomenon, with a decrease in the area of the medium grade and an increase in the area of the low and high grades. The three regions (the Loess Plateau, the Transition Zone, the West Qinling Mountains) showed a decreasing trend in moderately low grade and an increasing trend in moderately high grade as the area of the Loess Plateau within the region decreases. The three counties in the Transition Zone (ZXC, WSC, QZD) showed an increasing trend of the increase in low grade and a decreasing trend of the increase in moderately high grade as the area of the Loess Plateau within the county decreases.
3. From 2000 to 2020, the mean HQ in the study counties showed a trend of first increasing then decreasing, and HQ showed obvious spatial differentiation due to differences in the distribution of geomorphic types within study counties. With changes in geomorphic scale, HQ of the Loess Plateau, the Transition Zone, and the West Qinling Mountains showed a three‐level gradient of low, medium, and high. the Transition Zone (ZXC, WSC, QZD) generally showed the spatial distribution characteristics of low in the north and high in the south, and the low grade was mainly distributed in the Loess Plateau area within the county north. The formation of this geographical distribution further verified that the ecological effects of the Loess Plateau have influenced the HQ of the West Qinling Mountains.
4. LUI and the population density of human factors were the dominant factors causing spatial differentiation of HQ in the three regions, but with the national emphasis on environmental issues, the degree of human influence on HQ showed a tendency of first increasing then decreasing. NDVI and NPP of ecological factors have always played a key role in the variation of HQ in the Transition Zone, which vegetation cover significantly influenced the level of HQ in the Transition Zone. The interaction between ecological and human factors was stronger than the interaction within ecological factors and the interaction within human factors, so it is necessary to consider the effects of various factors comprehensively in the future to adopt differentiated strategies and improve regional HQ.

## Author Contributions

**Caihong Hui:** conceptualization (supporting), formal analysis (lead), methodology (lead), software (lead), writing – original draft (lead). **Xuelu Liu:** conceptualization (lead), funding acquisition (lead), supervision (lead), writing – review and editing (lead). **Miaomiao Zhang:** validation (lead), writing – review and editing (supporting). **Xiaoning Zhang:** data curation (lead), supervision (supporting). **Xingyu Liu:** methodology (supporting), software (supporting).

## Conflicts of Interest

The authors declare no conflicts of interest.

## Data Availability

The land use/cover data derived from the global 30 m land cover data (http://www.globallandcover.com), DEM data derived from Geospatial Data Cloud Platform with a spatial resolution of 30 m (http://www.gscloud.cn), mean annual temperature and annual rainfall data were obtained from the National Tibetan Plateau Science Data Centre (https://data.tpdc.ac.cn), normalized difference vegetation index (NDVI), net primary production (NPP), soil type, soil erosion, Gross domestic product (GDP), and nighttime light data were obtained from the Resource and Environmental Sciences Data Centre of the Chinese Academy of Sciences (http://www.resdc.cn). Population density was derived from the World Population Grid data (https://hub.worldpop.org).
